# Effect of Aqueous Extracts from *Solidago Canadensis* L. Leaves on Germination and Early Growth Stages of Three Cultivars of *Raphanus Sativus* L. Var. *Radicula* Pers

**DOI:** 10.3390/plants9111549

**Published:** 2020-11-12

**Authors:** Katarzyna Możdżeń, Beata Barabasz-Krasny, Peiman Zandi, Angelika Kliszcz, Joanna Puła

**Affiliations:** 1 Institute of Biology, Pedagogical University of Krakow, Podchorążych 2 St., 30-084 Kraków, Poland; katarzyna.mozdzen@up.krakow.pl (K.M.); beata.barabasz-krasny@up.krakow.pl (B.B.-K.); 2 Institute of Environmental and Sustainable Development in Agriculture, Chinese Academy of Agricultural Science, Beijing 100081, China; 2018y90100008@caas.cn; 3 Faculty of Agriculture and Economics, University of Agriculture in Kraków, Mickiewicz 21 Av., 31-120 Kraków, Poland; rrpula@cyf-kr.edu.pl

**Keywords:** decoction, germination indexes, infusion, macerate, radish, seedling

## Abstract

*Solidago canadensis* L. is an expansive perennial that forms persistent, species-poor plant communities. It often spreads in fallow areas, displacing native floristic ingredients. Its expansiveness is largely due to morphological features, but it can also be the effect of allelopathic interaction. The aim of the experiment was to investigate the effect of aqueous extracts (decoction, infusion, and maceration) from dry *S. canadensis* leaves on germination and early growth stages of *Raphanus sativus* L. var. *radicula* Pers., in three cultivars: ‘Rowa’, ‘Półdługa’, and ‘Krakowianka’. In comparison to the control, the percentage of germinated radish seeds of ‘Rowa’ cultivar was statistically lower on the infusion and macerate. Regardless of the cultivar, the smallest changes in germination were found in seeds watered with decoction, and the largest with macerate. Seedlings length was most inhibited on macerate substrates, and least with infusion. Regardless of the form of the extract, each of them negatively affected the initial growth of radish seedlings. A fresh mass of ‘Rowa’ seedlings was inhibited by all Canadian goldenrod extracts. In relation to the control, the ‘Krakowianka’ cultivar was the least sensitive to *S. canadensis* extracts. The total chlorophyll content was the lowest in the seedlings of the ‘Rowa’ and ‘Półdługa’ cultivars germinated on macerate, compared to the control and two others extracts. The percentage of electrolyte leakage depended on the type of extract used and the radish cultivar. The study showed that depending on the cultivar, the aqueous extracts from *S. canadensis* leaves decreasing of germination and early growth of *R. sativus*.

## 1. Introduction

The proliferation of invasive species, through competition, changes in resource availability, and disturbance to the ecosystem, is one of the greatest threats to biodiversity [1,2]. Due to the properties of easy adaptation to new habitat conditions, effective competition for space, water, nutrients and pollinating insects, they gradually displace autochthonous species [3,4,5,6,7,8]. A good example of this kind of competition is the Canadian goldenrod, a species easily populating new areas in various regions of the world [9].

**Figure 1:**
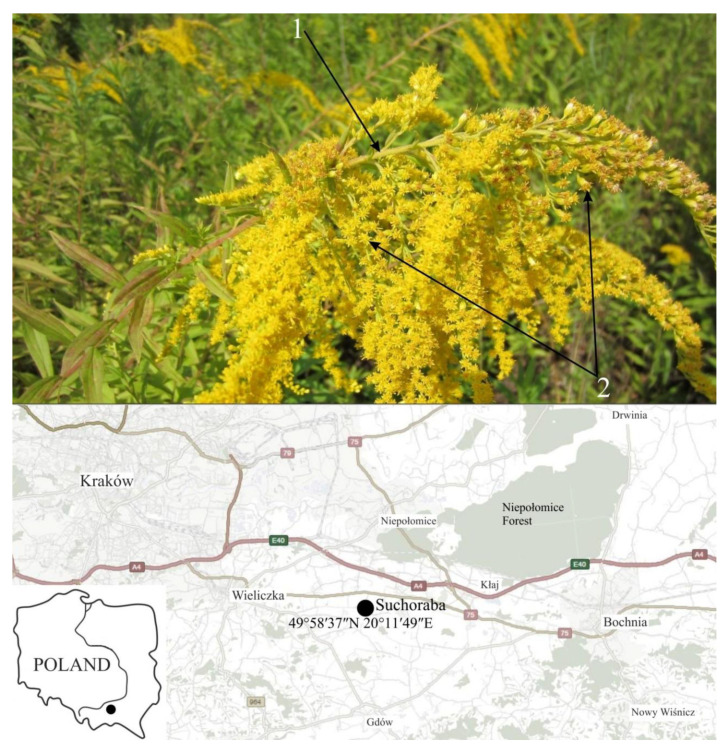
General appearance of *Solidago canadensis* L.–arrows indicate diagnostic features: (**1**) hairy stem and (**2**) all the “lingule” flowers of equal length in composite inflorescences (Photo. J. Puła); place of collecting the plant material for experiment.

Canadian goldenrod *Solidago canadensis* L. (Asteraceae) is a perennial herbaceous plant that originates in North America. Currently, it grows in Europe, Asia, Australia, and New Zealand. It is found in humid habitats, on meadows, roadsides, in ditches, on both shady and sunny areas [10]. It differs, from the similar *S. gigantea* Aiton, of hairiness of the stem up to the inflorescences and the same length of the tubular flowers in capitulas (*pseudanthia*) of the entire complex inflorescence, resembling a panicle (Figure 1). In Poland, *S. canadensis* was introduced for ornamental purposes and as a food source for bees. This species is currently found throughout Poland; only in the north-eastern part of its population is slightly smaller [9,11,12]. *S. canadensis* due to its high growth, ease of reproduction, shows strong competitive abilities and influences the distribution of native plant species [13,14,15].

According to Huang and Guo [16], *S. canadensis* significantly reduces the overall number and diversity of species and causes severe losses in agriculture [17,18,19]. For example, it reduces germination and growth of a mulberry (*Morus alba* L.); morning glory (*Pharbitis nil* (L.) Roth), wheat (*Triticum aestivum* L.) and rape (*Brassica campestris* (L.) Koch [17]. Researchers have offered various hypotheses to explain the increased competitive ability of invasive plant species. These include phenotypic plasticity, release from natural enemies, evolution of increased competitive ability and the production of allelopathic compounds. The effects of *S. canadensis* invasion are less in communities with more numbers of native species [18,20].

Abhilasha et al. [10] proved that *S. canadensis* releases allelochemical compounds into the soil. This invasive alien weed causes severe shifts in the microorganism community in invaded areas, e.g., the enhancement (number of nitrite bacteria, aerobic Azotobacter, sulfate reducer, ammonifier and aerobic cellulose-decomposer), as well as reduction in the growth of denitrifier anaerobic cellulose-decomposer and desulphate reducer [21]. Sometimes the presence of specific allelochemicals derived from *S. canadensis* could be suggested in tomatoes cultivation due to their inhibitory effect on tomatoes’ soilborne pathogens (i.e., *Pythium ultimum* Trow. and *Rhizoctonia solani* J.G. Kühn) [22]. Although other authors pointed out that *S. gigantea*’s effect on soil microorganisms cannot last in subsequent vegetation periods and it depends on occurring plants (i.e., plant communities). During the three years mesocosm experiment changes in measured soil enzymes (i.e., β-glucosidase, β-N-acetylhexosaminidase and acid phosphatase, the enzymes mediating mineralization of C, N and P, respectively) were not clear. Effecta of increased β-glucosidase activity in rgw second year were no longer visible in the third vegetation period. *S. gigantea* did not affect β-N-acetylhexosaminidase activity, and acid phosphatase activity increased in *Filipendulion* plant community, but decreased in *Molinion* and *Magnocaricion* plant communities [23]. Moreover, authors, based on fungal biomass content in the invaded soil (accumulated during experiment), concluded that *S. gigantea* in their invasion strategy seem to follow toward pathogen-free areas as the plants fail to control their fungal pathogens in place.

*S. canadensis’* main allelochemicals are flavones, phenolics, saponins, polyacetylates, essential oils [24,25], and other allelochemicals compounds, unknown so far [18,26]. Bearing in mind reports of allelopathic abilities of Canadian goldenrod, an attempt was made to assess this type of impact on seeds of *Raphanus sativus* L. var. *radicula* Pers. in three cultivars: ‘Rowa’, ‘Półdługa’, and ‘Krakowianka’. The research used radish seeds as a model organism. Radish seeds germinate in a very short time compared to other species. They have low nutritional and environmental requirements. As part of the research hypothesis, it was assumed that aqueous extracts of *S. canadensis* (in various forms: infusion, decoction and macerate) exhibit biological activity in relation to the studied radish cultivars, affecting: (1) its seed germination indexes, (2) length, fresh and dry mass of seedlings, (3) total chlorophyll content in seedlings and (4) percentage electrolytes leakage from seedling cells.

## 2. Results

### 2.1. Germination Indexes

On the first and second days of germination, the significantly highest germination speed (GS) of ‘Rowa’ and ‘Półdługa’ radish seeds was recorded in the control. On the third day, the lowest values of the index were recorded in all three radish cultivars on the macerate. In the following days, the GS was similar to seeds germinated on a distilled water and the decoction, regardless of the cultivar. In the case of the ‘Krakowianka’ cultivar, the highest GS for seeds watered with infusion was observed. For all radish cultivars treated with the macerate extract, a statistically significance decrease in GS values was found. On day 7, for the ‘Rowa’ and ‘Półdługa’ cultivars, the significantly highest percentage of germinated seeds was found for seeds treated with distilled water (control) and decoction. The macerate caused the complete disappearance of ‘Rowa’ cultivar germination. No radish sprouts were observed, but seeds were swollen, their seed coat was cracked in some cases. Significantly the lowest GS values for ’Półdługa’ and ’Krakowianka’ were found for seeds watered with macerate, compared to the control and the other two extracts (Table 1).

Compared to the control, the germination index (GI) values changed depending on the extract used. The largest changes were noted for macerate, in all radish cultivars. The decoction extract had the smallest but still statistically negative impact on the GI for ‘Rowa’ and ‘Krakowianka’. For the ‘Rowa’ and ‘Krakowianka’, no significance coefficient of the rate of germination (CRG) differences, between the control and decoction and infusion were observed. The CRG index for ‘Półdługa‘ was the highest for seeds watered with distilled water. For all radish cultivars germinated on macerate, the lowest values of this parameter were revealed. In the case of the T50 index, for ‘Rowa’ cultivar significant inhibiting influence of all *S. canadensis* aqueous extracts on the value of this parameter was demonstrated. For ‘Półdługa’, the infusion and macerate extracts decreased the T50, compared to the control. For ‘Krakowianka‘ a statistically significant differences were observed between macerate, and others extracts and distilled water. The seedling vigor index (SVI) index values for radish seedlings germinated on Petri dishes with aqueous extracts of *S. canadensis* were significantly lower, compared to the control. The smallest values of this parameter were shown for seedlings watered with macerate. The ‘Półdługa’ cultivar was an exception, for which no differences between the control and the macerate were found (Table 2).

### 2.2. Length of Seedlings

The growth of radish seedlings treated with aqueous extracts of Canadian goldenrod leaves was inhibited by all extracts. In comparison to the control, regardless the cultivar, the macerate had the most negative affected of seedlings length of radish (Table 3). The inhibition percentage (IP) values were the lowest for seedlings watered with infusion. Among the radish cultivars analyzed, the elongation was inhibited most in the ‘Rowa’ and in the least in ‘Krakowianka’ cultivars (Table 3).

### 2.3. Fresh, Dry Mass and Water Content

The fresh mass of radish seedlings was significantly larger in the control conditions for ‘Rowa‘ and ‘Półdługa‘. For ‘Rowa‘ cultivar, each of the type extracts decreased the fresh mass values. On the macerate extracts, ‘Rowa‘ seeds was swollen and had a crack seed coat, which significantly influenced the lowering of this parameter. The fresh mass of ‘Półdługa‘ seedlings was inhibited by decoction and macerate, compared to the control. For the ‘Krakowianka‘ no statistical differences in the values of this parameter were observed. Generally, the largest inhibition of fresh mass values were revealed for radish seedlings on macerate (Table 4).

The dry mass values of all radish cultivars germinated on *S. canadensis* extracts, did not differ significantly, compared to the control. The percentage of water content in ‘Rowa’ radish seedlings was significantly lower for seedlings watered with macerate. For the ‘Półdługa’ and ‘Krakowianka’ cultivars, no statistically significant changes were found in the values of this parameter (Table 4).

### 2.4. Chlorophyll Content and Electrolyte Leakage

**Figure 2:**
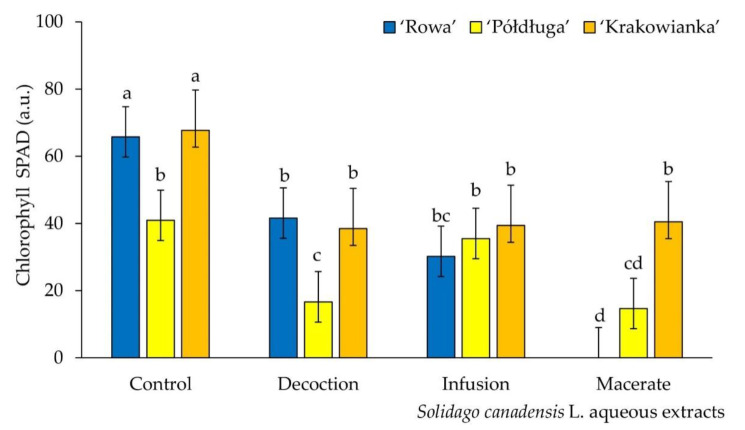
Chlorophyll content of radish seeds (*Raphanus sativus* L. cv. Rowa, Półdługa, Krakowianka) treatment of aqueous extracts from *Solidago canadensis* L. leaves; mean values ± SD (*n* = 10) with different letters (a–d) differ statistically according to Duncan test at *p* < 0.05.

The total chlorophyll content in seedlings of ‘Rowa‘ and ‘Krakowianka‘ was significantly lower in each treatment, compared to the control sample. The lowest concentration of chlorophyll was demonstrated for seedlings of the ‘Rowa’ and ‘Półdługa’ cultivars, watered with macerate. For the ‘Półdługa‘ cultivar, no differences in the chlorophyll content were found between the seedlings grown on the infusion and distilled water (Figure 2).

**Figure 3:**
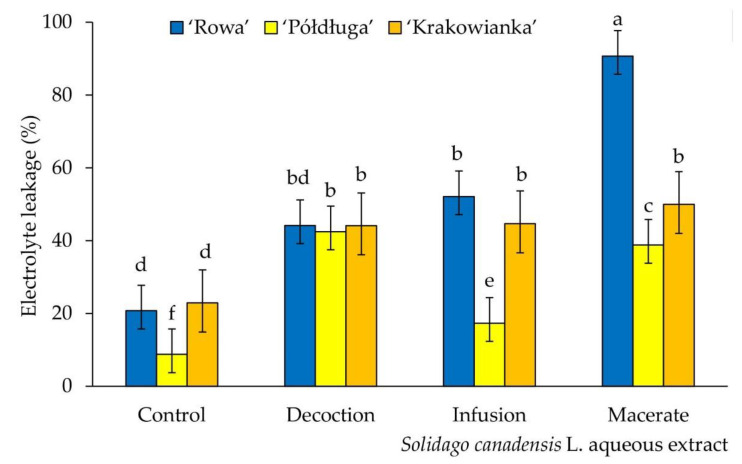
Electrolyte leakage of three cultivars (‘Rowa‘, ‘Półdługa‘, ‘Krakowianka‘) of radish seeds (*Raphanus sativus* L.) treatment of aqueous extracts from *Solidago canadensis* L. leaves; mean values ± SD (*n* = 10) with different letter (a–f) differ statistically according to Duncan test at *p* < 0.05.

Percentage of electrolytes’ leakage in radish seedlings was the smallest in the control. Each of the aqueous extracts increased the degree of destabilization of the cell membranes of seedlings, regardless of the cultivar. The largest disturbances in water and ion management were found in seedlings of the ‘Rowa’ cultivar watered with macerate, in comparison to tested cultivars and extracts type. The ‘Rowa’ seeds on macerate, unlike the other varieties and extracts, were swollen, their seed coat was cracked in some case, hence most probably such a high% of electrolyte leakage. Compared to the control, for the ‘Rowa’ cultivar watered with decoction, no statistically significant differences were found. However, for the other two cultivars, significant differences were observed in the values of this parameter (Figure 3).

## 3. Discussion

In the conducted studies, aqueous extracts of *Solidago canadensis* L. leaves significantly slowed germination of seeds of the analyzed radish cultivars. Generally, germination indexes in each of the types of extracts were significantly lower, compared to the control (Table 1 and Table 2). The highest sensitivity to the effect of the extracts was demonstrated for the ‘Rowa’, and the lowest for ‘Krakowianka’ cultivar. Confirmation for the results obtained here, indicating a strong inhibitory effect of chemicals released from goldenrod leaves, are previous experimental studies [27,28,29,30]. Allelopathic potential of goldenrod extracts was found, among others in crops of carrot (*Daucus* sp.), barley (*Hordeum* sp.), maize (*Zea mays* L.) [31], lettuce (*Lactuca* sp.) [32] and coriander (*Coriandrum* sp.). The effect was observed even for crop’s weeds such as: velvetleaf (*Abutilon theophrasti* Med.) and redroot pigweed (*Amaranthus retroflexus* L.) [33].

The consequence of the negative impact of Canadian goldenrod on the germination of radish seeds was the inhibition of the elongation growth of seedlings and a decrease in the value of fresh mass. In the case of the dry mass, a statistically significant differences was not noted for tested radish cultivars, watered with three types of extracts (Table 3 and Table 4). Bingyao et al. [17] and Butcko and Jensen [34] observed the inhibitory and stimulating effect of *S. canadensis* extracts. The positive effect on mass increase may result from the stimulating effect of growth regulators responsible for cell growth, or from the activity of enzymes that improve metabolism and accelerate the flow of leaf assimilates to spare organs [35,36].

Aqueous extracts from goldenrod also decreased the chlorophyll content (Figure 2). Chlorophyll is one of the chemical compounds contained in plants that affects their photosynthetic activity and, consequently, the production of biomass. In chloroplasts, it is associated with specific proteins, glycolipids and sulfolipids. Its low content may be caused by inhibition of the synthesis of magnesium and iron chelate and protoporphyrinogen IX [37,38]. Observations of changes in chlorophyll content carried out here were significantly related to the type of extract used and the radish cultivar. In these measurements, the ‘Rowa’ cultivar was the most sensitive to the macerate extract. It can be assumed that the observed negative effects of using goldenrod extracts may be an effect of allelochemical compounds. Plants subjected to allelopathy stress vary in color intensity, which shows that they most likely contain less chlorophyll [39]. The chlorophyll content in the leaves is reduced, e.g., by phenolics [40]. The effect of allelochemical inhibitors depends on their concentration in the extract. The reduction of chlorophyll content may be caused by its degradation or may be a consequence of the restriction of its synthesis by allelochemical substances [37,41].

Each of the types of extracts used also caused an increase in the electrolyte leakage, which disrupted the water-ion balance of radish seedlings (Figure 3). Cell membranes are complex and dynamic structures made of lipids and proteins [42]. They are a measure of structural integrity, function stability and an indicator of plant tolerance to stress. Membranes are one of the first structures affected by stress factors. Their mechanism of functioning and physiological role indicate that electrolyte leakage is associated with the loss of K^+^ ions, mediating the conductivity of cations through the membrane [43]. In the conducted studies, it was found that the extract prepared in the form of macerate, irrespective of the radish cultivar, caused the largest inhibitory effect for the leakage of electrolytes. In the wide spectrum of allelochemical compounds, such properties are attributed to phenolic compounds [44]. These compounds are among the most common synthesized by plants and show a negative effect even at low concentrations. Allelochemical substances can penetrate into the cell membranes and destroy their structural connections or create new modification. For example, phenolic acids can change channels structure in cell membranes and limit the functioning of enzyme proteins, ATP, effect on transport and accumulation of ions between membranes and the water management of the cell. Other compounds, e.g., terpenoids, quinones, p-coumaric acid and ferulic acid cause lipid peroxidation. The cell membranes are often damaged by free radicals, which are formed, among others, by during the oxidative transformation of phenolics [44,45,46,47].

Differences in germination and early growth of radish seedlings in the presence of aqueous extracts from *S. canadensis* showed specificity of seed responses. They depended on both the radish cultivar and the type of extract used. Essentially, macerates had the most negative effects on germination and seed growth as well as chlorophyll content and electrolyte leakage. Extracts in the form of decoction and infusion also limited these processes, but to a much lesser extent than macerate. This kind of plant structure response suggests that aqueous extracts from Canadian goldenrod dry leaves in all forms have allelopathic properties. The leaves, from mature and flowering individuals used in the experiment, had a clearly inhibitory effect on the physiology of radish seeds. According to earlier studies on allelopathy [48], this may also suggest that leaves from young plants have even greater allelopathic potential. Therefore, the hypothesis on the biological properties of Canadian goldenrod can be here clearly confirmed. *S. canadensis* owes its expansion, as an invasive species, not only to clonal growth and interspecies relations [20], but also to the production of allelochemical compounds that significantly affect soil microflora and other plants [4,10,17,49].

The emission of allelochemical substances to the soil by species of the genus *Solidago* may pose a significant threat in the restoration of wasteland for agricultural production [50]. Therefore, one of the tasks in combating invasive weeds, such as goldenrod, is to clarify their adaptation to the environment, both physiologically and ecologically [51]. This will help to introduce effective and synthetic schemes for their elimination, forecast potential distribution areas and create a system for estimating threats to other species [52]. In Poland, no activities have been carried out to control and eliminate goldenrod so far. The only treatments used in the practice of controlling *Solidago* are regular mowing, point destruction of plants by pulling or digging, as well as afforestation, which increases shading and worsens habitat conditions for goldenrod [6,9]. Therefore, further research is needed to find tolerant and susceptible crops to goldenrod to fully assess its allelopathic properties.

## 4. Materials and Methods

### 4.1. Plant Material

*Solidago canadensis* L. leaves were collected during the flowering phase at the turn of July and August 2019, in Suchoraba (49°58′37″ N 20°11′49″ E) in southern Poland (Figure 1). The species occurred there massively, in the form of a compact yield, in a fallow field. The harvested fresh Canadian goldenrod leaf material was sorted such that pure plant material was selected for drying. Then the leaves were placed in a single layer on sheets of filter paper and dried in the dark, in an dryer at a constant temperature of 25 °C (Wamed SUP-100, Warsaw, Poland) for 7 days. Properly dried leaves had a natural color and crumbled easily. The dried plant material was stored in a silica gel desiccator.

Radish seeds were purchased at “Polan”–Breeding and Seed Horticulture in Krakow (Poland) company.

### 4.2. Extract Preparation

The dried Canadian goldenrod leaves were ground mechanically in a mill (Braun, 4045 type, Germany), and then aqueous extracts in the form of decoction, infusion and macerate were prepared, according to Tyszyńska-Kownacka and Starek [53]. The infusion was prepared from 5 g of dry and crushed *S. canadensis* leaves, which were flooded over with 250 mL of boiling distilled water and left covered for 30 min. After cooling, the extract was filtered through Whatman filter papers. The decoction was prepared from 8.75 g of dry plant material poured with a 1 liter of cold distilled water. The solution was mixed and left for 24 h in the dark, at 20–25 °C temperature. Then, the extract was boiled for 15 min and filtered. A macerated extract was prepared by flooding 5 g of dry leaves of 100 mL cold distilled water and left in the dark, at room temperature for 24 h. After one day, the extract was filtered the same form as infusion and decoction. The extracts were stored during the experiment at 8 °C.

### 4.3. Germination Conditions

Seeds of radish cultivars (*Raphanus sativus* L. cv. Rowa, Półdługa and Krakowianka) were purified in 5% sodium hypochlorite by 5 min and then washed three times with distilled water. The radish seeds (25 seeds per dish) were placed on sterile Petri dishes (ø 9 cm) with three layers of Whatman filter paper (Grade 1: 11 μm–medium flow filter paper). At the beginning of the experiment, the seeds were watered with the aqueous extracts (6 mL) in different form: decoctions, infusions, and macerate from *S. canadensis* leaves. A control group was seeds watered with distilled water. Every day seeds were watered with 3 mL of extracts and distilled water (control group). The Petri dishes with seeds were placed in a growth chamber (Angelantoni Industrie, Massa Martana, Italy) at 12 h/12 h (day/night) photoperiod, at a light intensity of 200 μmol × m^–2^ × s^–1^; the temperature was 25 °C/20 °C, and the relative humidity oscillated around 70–90%.

### 4.4. Germination Indexes

The germination capacity of seeds were checked every day (by 7 days). After this time, the following germination indexes were determined: a germination rate (GR), and germination speed (GS) [54], seedling vigor index (SVI) [55], coefficient of the rate of germination (CRG) [56], time required for 50% germination (T50) [57].

GR = (number of germinated seeds/total number of seeds) × 100,(1)

GS = ((GT × D)/(GC × D)) × 100,(2)

where: GT is the number of germinated seeds daily in the treatment, GC is the number of germinated seeds daily in the control, and D is the number of corresponding days.

SVI = (seedling length (cm) × percentage of germinated seeds)/100,(3)

CRG = ((n1 + n2 + nn)/((n1 × T1) + (n2 × T2) + (n3 × T3) + …)) × 100,(4)

where: n1 = number of germinated seeds on time T1; n2 = number of germinated seeds on time T2; n3 = number of germinated seeds on timeT3.

T50 = ti + ((N/2) − nj) × (ti − tj))/(ni − nj),(5)

where: N is the final number of germination and ni, nj cumulative numbers of seeds germinated by adjacent counts at times ti and tj when ni < N/2 < nj.

### 4.5. Seedlings Length

Effects of aqueous extracts from *S. canadensis* leaves on seedlings growth (length of under-and above ground parts) were measured by a caliper (TOPEX 31C615, Poland), with an accuracy of 0,1 cm, after 7 days of experiment. An inhibition percentage expressed as % index (IP) was measured according to formula used by Islam and Kato-Noguchi [58].

IP = (1 − (L_E_/L_C_)) × 100, (6)

where L_E_—seedling length (cm) treated with the aqueous extract, L_C_—seedling length (cm) treated with the distilled water (control group).

### 4.6. Fresh, Dry Mass and Water Content

After 7 days of germination the values of fresh and dry masses of whole seedlings (together under–and aboveground parts) were measured. Seedlings were weighted (fresh mass–FM) (Radwag WPS120, Radom, Poland) and dried (dry mass–DM) at 105 °C for 48 h in dryer (Wamed SUP 100, Warsaw, Poland). The tissue water content (% H_2_O) was determined according to the formula % H_2_O = 100 − ((DM × 100)/FM).

### 4.7. Chlorophyll Content and Electrolyte Leakage

The total chlorophyll content was measured by Chl SPAD by inserting the cotyledon into the clip of the SPAD chlorophyllometer device. It is not invasive method. The electrolyte leakage in radish seedlings was measured according to method used by Redmann et al. [59]. After 7 days of germination, single seedlings were placed in polypropylene vials with 30 mL distilled water and shaken for 3 h on a shaker (Labnet, Rocker, New Jersey, NJ, USA) to determine the electrolytes leakage from live cells (E1). The electrolytes leakage was measured by conductivity meter (type CX-701) with electrode (K = 0.92) (Elmetron, Zabrze, Poland). Then the plant material in vials was frozen at −70 °C. After 24 h the shaking procedure was repeated to the total electrolyte leakage from dead cells (E2) was measured. The percentage of electrolyte leakage (EL) was calculated according to the formula: EL = (E1/E2) 100%. The obtained percent results of electrolyte leakages from the control group of seedlings were compared to the values of seedlings germinated on the dishes with aqueous extracts.

### 4.8. Statistic Analysis

The experiment was carried out in three repetitions in two independent series. Significance of differences between objects (means ± SD; *n* = 10) was tested using the ANOVA multifactor analysis test, Duncan post hoc at *p* < 0.05 in Statistica 13.0 for Windows.

## 5. Conclusions

In the studies carried out here, the impact of aqueous extracts was uneven and clearly depended on the type of acceptor (radish cultivar) and donor (extract types).

(1) After seven days, the germination speed of seeds was the highest for cultivars watered with decoction and infusion, compared to the control. The macerate caused the complete disappearance of ‘Rowa’ germination. On the *Solidago canadensis* decoction radish seeds germinated better than on macerate extracts (most similar to the natural source of allelochemical compounds from dead organic debris). The values of germination indexes differed depending on the cultivar. The macerate extract showed a significant reduction in the value of germination indexes for all radish cultivars, in relation to the control. (2) The longest seedlings among all radish cultivars, grown on distilled water, were observed. The most inhibiting properties had a macerate. Regardless of the form of leaves extracts, each of them inhibited the elongation of seedlings of three radish cultivars, in comparison to the control group. The fresh mass of ‘Rowa’ seedlings were significant reduced by each of the *S. canadensis* extracts. The dry mass for all radish cultivars by *S. canadensis* extracts was not differ significantly, compared to the control values. (3) The all aqueous extracts from *S. canadensis* leaves, for ‘Rowa’ and ‘Krakowianka‘ cultivars, reduced the chlorophyll content. (4) Compared to the control, with the ‘Rowa’ cultivar the highest electrolyte leakage on macerate from *S. canadensis* leaves was revealed. All extracts caused an increase the electrolyte leakage in three cultivars of radish seedlings. The exception was the ‘Rowa’ cultivar treated to decoction, which no statistically significant differences were found in the destabilization of cell membranes.

In the context of these studies and hypothesis, it can be assumed that *S. canadensis* contributes to the delayed germination process of radish seeds, already in the early stages of vegetation.

## Figures and Tables

**Table 1:** Germination speed (GS) (%) of *Raphanus sativus* L. cv. Rowa, Półdługa, Krakowianka, on the aqueous extracts from *Solidago canadensis* L. leaves and distilled water (control).

Treatment	Days (24 h)						
	1	2	3	4	5	6	7
	‘Rowa’						
Control	73.16 ^a^ ± 1.52	75.00 ^b^ ± 1.67	75.00 ^c^ ± 2.83	75.00 ^c^ ± 2.17	80.00 ^b^ ± 0.00	84.60 ^b^ ± 0.00	92.80 ^ab^ ± 0.00
Decoction	38.24 ^b^ ± 1.14	49.96 ^c^ ± 1.22	74.08 ^c^ ± 5.93	86.6 ^bc^ ± 1.58	92.68 ^ab^ ± 1.30	94.40 ^ab^ ± 1.34	92.68 ^ab^ ± 1.14
Infusion	13.92 ^d^ ± 0.55	20.32 ^d^ ± 1.22	23.52 ^d^ ± 0.84	28.32 ^e^ ± 0.71	33.12 ^e^ ± 0.84	35.52 ^e^ ± 0.84	37.84 ^d^ ± 0.89
Macerate	0.00 ^e^ ± 0.00	0.00 ^f^ ± 0.00	0.00 ^e^ ± 0.00	0.00 ^f^ ± 0.00	0.00 ^f^ ± 0.00	0.00 ^f^ ± 0.00	0.00 ^e^ ± 0.00
	‘Półdługa’						
Control	77.52 ^a^ ± 1.87	100.00 ^a^ ± 1.14	100.00 ^a^ ± 2.00	100.00 ^a^ ± 1.79	100.00 ^a^ ± 1.34	100.00 ^a^ ± 0.00	100.00 ^a^ ± 0.00
Decoction	38.28 ^b^ ± 0.71	46.28 ^c^ ± 0.71	83.88 ^bc^ ± 3.13	100.00 ^a^ ± 2.28	100.00 ^a^ ± 0.89	100.00 ^a^ ± 0.00	100.00 ^a^ ± 0.00
Infusion	32.76 ^b^ ± 1.14	40.76 ^c^ ± 0.89	73.56 ^c^ ± 2.30	86.36 ^bc^ ± 2.77	95.16 ^ab^ ± 2.55	100.00 ^a^ ± 2.30	100.00 ^a^ ± 2.07
Macerate	12.88 ^d^ ± 0.00	12.88 ^e^ ± 0.00	20.88 ^d^ ± 0.71	29.68 ^e^ ± 1.30	49.68 ^d^ ± 3.56	62.48 ^d^ ± 4.34	72.88 ^c^ ± 5.34
	‘Krakowianka’						
Control	74.80 ^a^ ± 0.89	86.00 ^b^ ± 1.14	92.40 ^b^ ± 0.00	92.40 ^b^ ± 0.00	92.40 ^ab^ ± 0.00	92.40 ^ab^ ± 0.00	92.40 ^ab^ ± 0.00
Decoction	77.12 ^a^ ± 0.71	81.12 ^b^ ± 1.14	81.12 ^bc^ ± 0.89	81.12 ^bc^ ± 1.22	81.12 ^b^ ± 0.55	81.12 ^b^ ± 0.45	81.12 ^b^ ± 0.45
Infusion	77.24 ^a^ ± 2.77	86.04 ^b^ ± 4.00	93.24 ^b^ ± 4.38	100.00 ^a^ ± 3.91	100.00 ^a^ ± 2.61	100.00 ^a^ ± 2.17	100.00 ^a^ ± 0.45
Macerate	22.12 ^c^ ± 0.84	30.92 ^cd^ ± 1.30	40.92 ^c^ ± 1.79	51.32 ^d^ ± 1.95	63.32 ^c^ ± 2.59	76.52 ^c^ ± 1.87	82.12 ^b^ ± 1.87

^(a–f)^ mean values ± SD (*n* = 10) with different letters differ (in column) statistically according Duncan test at *p* < 0.05.

**Table 2:** Germination parameters of *Raphanus sativus* L. cv. Rowa, Półdługa, Krakowianka seeds germinated on the aqueous extracts from *Solidago canadensis* L. leaves and distilled water (control).

*Raphanus sativus* Cultivars	Treatment	GI	CRG	T50	SVI
‘Rowa’	Control	52.70 ^b^ ± 2.17	20.71 ^b^ ± 0.25	0.47 ^a^ ± 0.001	1.75 ^b^ ± 0.66
	Decoction	30.81 ^c^ ± 4.17	18.31 ^b^ ± 0.56	0.45 ^b^ ± 0.001	0.40 ^c^ ± 0.15
	Infusion	9.80 ^e^ ± 1.00	18.46 ^b^ ± 0.45	0.31 ^d^ ± 0.04	0.20 ^c^ ± 0.03
	Macerate	0.00 ^f^ ± 0.00	0.00 ^d^ ± 0.00	0.00 ^e^ ± 0.00	0.00 ^d^ ± 0.00
‘Półdługa’	Control	55.76 ^b^ ± 2.88	21.05 ^ab^ ± 0.28	0.46 ^a^ ± 0.001	1.64 ^b^ ± 0.39
	Decoction	57.03 ^b^ ± 2.29	18.17 ^c^ ± 0.33	0.46 ^a^ ± 0.001	0.77 ^c^ ± 0.12
	Infusion	25.70 ^ab^ ± 3.84	17.98 ^c^ ± 0.35	0.45 ^b^ ± 0.01	0.76 ^c^ ± 0.27
	Macerate	9.79 ^e^ ± 3.40	15.64 ^c^ ± 0.21	0.40 ^c^ ± 0.08	1.62 ^bc^ ± 0.01
‘Krakowianka’	Control	62.75 ^a^ ± 1.44	21.74 ^b^ ± 0.15	0.46 ^a^ ± 0.001	4.59 ^a^ ± 1.57
	Decoction	57.03 ^b^ ± 1.44	21.95 ^ab^ ± 0.15	0.46 ^a^ ± 0.001	1.95 ^b^ ± 0.50
	Infusion	54.21 ^b^ ± 7.71	20.92 ^ab^ ± 0.68	0.46 ^a^ ± 0.001	2.26 ^b^ ± 0.97
	Macerate	16.16 ^d^ ± 1.99	18.08 ^c^ ± 0.51	0.41 ^bc^ ± 0.02	0.82 ^c^ ± 0.13

^(a–f)^ mean values ± SD (*n* = 10) with different letters differ (in column) statistically according Duncan test at *p* < 0.05; GI–germination index, CRG–coefficient of the rate of germination, T50–time required for 50% germination, SVI–seed vigor index.

**Table 3:** Length of *Raphanus sativus* L. cv. Rowa, Półdługa, Krakowianka seedlings, treatment of aqueous extracts from *Solidago canadensis* L. leaves.

*Raphanus sativus* Cultivars	Control (cm)	Decoction		Infusion		Macerate	
		(cm)	IP (%)	(cm)	IP (%)	(cm)	IP (%)
‘Rowa’	7.10 ^c^ ± 2.63	1.70 ^f^ ± 0.66	70.57	2.64 ^e^ ± 0.38	56.65	0.00 ^g^ ± 0.00	100.00
‘Półdługa’	6.56 ^c^ ± 1.58	3.08 ^d^ ± 0.48	50.55	3.50 ^d^ ± 1.17	44.48	2.14 ^e^ ± 1.25	69.50
‘Krakowianka’	13.06 ^a^ ± 2.16	7.86 ^c^ ± 1.94	39.02	9.14 ^b^ ± 3.99	27.54	6.06 ^c^ ± 1.44	51.83

^(a–g)^ mean values ± SD (*n* = 10) with different letters differ statistically according Duncan test at *p* < 0.05.IP–inhibition percentage expressed as % of control, minus (−) values of IP indicates growth stimulation, and plus (+) values of IP indicates growth inhibition.

**Table 4:** Fresh and dry mass and water content of radish seeds *Raphanus sativus* L. cv. Rowa, Półdługa, Krakowianka, treatment of aqueous extracts from *Solidago canadensis* L. leaves.

*Raphanus sativus* Cultivars	Control	Decoction	Infusion	Macerate
	Fresh Mass (mg)			
‘Rowa’	106.8 ^a^ ± 0.02	50.2 ^b^ ± 0.01	58.0 ^b^ ± 0.01	14.6 ^c^ ± 0.01
‘Półdługa’	116.2 ^a^ ± 0.06	48.4 ^b^ ± 0.01	67.8 ^ab^ ± 0.02	46.0 ^b^ ± 0.01
‘Krakowianka’	112.3 ^a^ ± 0.06	96.2 ^a^ ± 0.02	92.2 ^a^ ± 0.02	91.4 ^a^ ± 0.01
	Dry mass (mg)			
‘Rowa’	5.0 ^b^ ± 0.002	4.8 ^bc^ ± 0.003	7.8 ^ab^ ± 0.003	7.2 ^ab^ ± 0.003
‘Półdługa’	5.6 ^ab^ ± 0.002	5.4 ^ab^ ± 0.002	7.0 ^ab^ ± 0.002	7.4 ^ab^ ± 0.001
‘Krakowianka’	8.4 ^a^ ± 0.003	7.0 ^ab^ ± 0.001	6.4 ^ab^ ± 0.002	8.6 ^a^ ± 0.002
	Water content (%)			
‘Rowa’	95.30 ^a^ ± 2.35	90.91 ^a^ ± 4.55	86.28 ^a^ ± 5.78	50.85 ^c^ ± 11.08
‘Półdługa’	95.23 ^a^ ± 1.38	88.09 ^a^ ± 6.19	89.62 ^a^ ± 0.67	83.79 ^a^ ± 1.94
‘Krakowianka’	77.91 ^b^ ± 3.87	92.66 ^a^ ± 1.21	92.98 ^a^ ± 1.61	90.63 ^a^ ± 1.75

^(a–c)^ mean values ± SD (*n* = 10) with different letter differ statistically according to Duncan test at *p* < 0.05.
